# Whole Genome Sequencing of Methicillin-Resistant *Staphylococcus epidermidis* Clinical Isolates Reveals Variable Composite SCC*mec* ACME among Different STs in a Tertiary Care Hospital in Oman

**DOI:** 10.3390/microorganisms9091824

**Published:** 2021-08-27

**Authors:** Zaaima Al-Jabri, Zahra AL-Shabibi, Atika AL-Bimani, Amal AL-Hinai, Ammar AL-Shabibi, Meher Rizvi

**Affiliations:** 1 Department of Microbiology & Immunology 1, College of Medicine and Health Sciences, Sultan Qaboos University, Muscat 123, Oman; bimani2012@squ.edu.om (A.A.-B.); amhinai@squ.edu.om (A.A.-H.); s119848@sultanqaboosuniversity.onmicrosoft.com (A.A.-S.); rizvimeher@squ.edu.om (M.R.); 2 Oman Animal and Plant Genetic Resources Centre, Ministry of Higher Education, Research and Innovation, Muscat 112, Oman; Zahra-alshabibi@hotmail.com

**Keywords:** *Staphylococcus epidermidis*, mobile genetic element, ACME, COMER, SCC*mec*

## Abstract

*Staphylococcus epidermidis* has been recently recognized as an emerging nosocomial pathogen. There are concerns over the increasing virulence potential of this commensal due to the capabilities of transferring mobile genetic elements to *Staphylococcus aureus* through staphylococcal chromosomal cassette (SCC*mec*) and the closely related arginine catabolic mobile element (ACME) and the copper and mercury resistance island (COMER). The potential pathogenicity of *S. epidermidis*, particularly from blood stream infections, has been poorly investigated. In this study, 24 *S. epidermidis* isolated from blood stream infections from Oman were investigated using whole genome sequence analysis. Core genome phylogenetic trees revealed one third of the isolates belong to the multidrug resistance ST-2. Genomic analysis unraveled a common occurrence of SCC*mec* type IV and ACME element predominantly type I arranged in a composite island. The genetic composition of ACME was highly variable among isolates of same or different STs. The COMER-like island was absent in all of our isolates. Reduced copper susceptibility was observed among isolates of ST-2 and ACME type I, followed by ACME type V. In conclusion, in this work, we identify a prevalent occurrence of highly variable ACME elements in different hospital STs of *S. epidermidis* in Oman, thus strongly suggesting the hypothesis that ACME types evolved from closely related STs.

## 1. Introduction

*Staphylococcus epidermidis*, despite being a member of the skin normal flora, has been increasingly associated with bacteremias, skin and soft tissue infections, and device-associated infections [1,2]. In a recent study, a collection of 283 *S. epidermidis* whole genome sequences of a worldwide data set were analyzed to validate the distribution of the composite *Staphylococcus aureus* chromosomal cassette elements (SCC*mec*) elements [3]. The methicillin-resistant *S. epidermidis* (MRSE) lineage mostly belonged to three groups: two clusters of sequence type-2 (ST-2), and one ST-23 [4], supporting the concerns raised in numerous smaller scale and local studies that *S. epidermidis* is a dominant reservoir of multidrug resistance genes through horizontal gene transfer (HGT) [4,5]. The MRSA emerging strain USA300 in North America as well as *S. epidermidis* ATCC 12228 harbor two mobile genetic elements—the arginine catabolic mobile element (ACME) and the copper and mercury resistance island (COMER), which are closely related to SCC*mec* forming composite islands [6,7]. The mosaic composition of ACME elements is attributed to the presence of internal direct repeats (DR) allowing mobilization of heavy metal conferring genes and formation of conserved modules [8]. ACME elements are thought to contribute to the persistence of *S. epidermidis* as a colonizer on the skin [6,9]. Five main types of ACME have been identified in *S. epidermidis* so far, based on the presence and or absence of three main operons, namely *arc*, *opp3*, and *kdp*, as with different flanking DRs [10,11]. 

COMER was first described in *S. epidermidis* recently, next to SCC*mec* type IV [1]; it confers hyper-resistance phenotype to copper, resulting in enhancement of the fitness within the host macrophages in *S. aureus* [6,12,13]. However, in a recent study, COMER-like elements had no significant reduced susceptibility to copper in *S. epidermidis*. Copper is a key element acting as a cofactor for several key enzymes in the bacteria. During the infection, the innate immunity of the host responds by accumulating copper to kill the invading micro-organisms [14,15,16,17,18]. In *S. aureus*, the COMER element is found in MRSA-SAE as replacing the ACME element in MRSA-NAE and is composed of *merR/A/B* genes and the *cop* operon [7,13]. The COMER element demonstrates high genomic stability due to a significantly lower excision rate of SCC*mec* compared to ACME in *S. epidermidis* [3,19,20]. Nevertheless, the *copB* locus is still uniquely shared between ACME and COMER; thus, this phenomenon emphasizes the role of *copB* in maintaining copper homeostasis/resistance in staphylococcal species [7,21].

In this study, we examine the composition of ACME, antimicrobial resistance, and biocide resistance determinants in 24 *S. epidermidis* bacteremia isolates from Oman to expand our knowledge in the copper efflux systems and appreciate the variability of our epidemiology compared to the global pattern. To our knowledge, this is the first study addressing the role of ACME element in the spread of resistance determinants in *S. epidermidis* isolated from bloodstream infections in Oman using whole genome sequencing (WGS) analysis. Based on our genomic data from the current study, we aim to take this further and analyze the biocide resistance genes and whether their epidemiology supports or refute the heavy metal resistance genes related to SCC*mec* elements.

## 2. Materials and Methods

### 2.1. Bacterial Isolates

We investigated 24 *S. epidermidis* clinical isolates from blood culture samples of patients admitted to Sultan Qaboos University Hospital (Muscat, Oman) between July 2018 to January 2019 processed in the microbiology and immunology laboratory. Colonies from purity plates were used to make frozen stock of our samples in cryotubes containing beads according to the manufacturer’s instructions (Mast Diagnostics, Derby, UK). The samples were frozen at −80 °C for future use.

### 2.2. Genomic DNA Extraction and Whole Genome Sequencing (WGS)

The isolates were streaked on tryptic soy agar (TSA) plates (Oxoid, Basingstoke Hampshire, UK) and incubated at 37 °C for 16–24 h. Single colonies were then subcultured in 5 mL TSB and incubated overnight at 37 °C/200 rpm in a shaking incubator (Innova 4000, New Brunswick Scientific, Hertfordshire, UK). Prior to extraction, 20–40 mg of pelleted bacterial cells samples were pretreated within a previously prepared 100 μL of 0.1 mg/mL pre-lysis buffer containing lysozyme and lysostaphin (Thermo Fisher Scientific, Winsford, UK) and incubated for 30 min at 37 °C for complete lysis of the cell wall. Genomic DNA was extracted from bacterial colonies using commercial kits as per the manufacture’s protocols and eluted in 30 μL of molecular grade water (QIAamp^®^ genomic DNA kit, Hilden, Germany). Genomic DNA for all samples was quantified using NanoDrop 2000c spectrophotometer (Thermo Fisher Scientific) to ensure that adequate and pure DNA samples were obtained. The integrity of the DNA samples was checked using gel electrophoresis. The DNA was sent to the sequencing facility MicrobesNG (Birmingham, UK) for whole genome sequencing (WGS). Standard sequencing service was performed on the Illumina sequencing platform for all samples. Assembled and annotated contigs were analyzed using bioinformatics tools as described on the company’s website as follows: Genomic DNA libraries were prepared using the Nextera XT Library Prep Kit (Illumina, San Diego, CA, USA) following the manufacturer’s protocol with the following modifications: input DNA was increased twofold, and PCR elongation time was increased to 45 s. DNA quantification and library preparation were carried out on a Hamilton Microlab STAR automated liquid handling system (Hamilton, Bonaduz GA, Switzerland). Pooled libraries were quantified using the Kapa Biosystems Library Quantification Kit for Illumina. Libraries were sequenced using Illumina sequencers (HiSeq/NovaSeq) using a 250 bp paired end protocol. Reads were adapter trimmed using Trimmomatic 0.30 with a sliding window quality cutoff of Q15 [22,23,24]. A de novo assembly of the reads was performed using SPAdes version 3.2 [25], and the reads were mapped back to the resultant contigs, again using BWA mem to get more quality metrics. Using the most suitable reference, variants were predicted relative to the reference. Variant calling was performed using VarScan [26]. An automated annotation was performed using Prokka [27]. Details of WGS data of *S. epidermidis* including coverage, trimmed reads, taxonomic distribution, and assemblies can be found in Table S1 in the Supplementary Materials.

### 2.3. Mutlilocus Sequence Typing (MLST) and Whole Genome Single Nucleotide Polymorphism (SNP) Phylogeny Tree

Mutlilocus sequence typing (MLST) was determined for each *S. epidermidis* isolate using the Center for Genomic Epidemiology MLST database [27]. A whole genome SNP alignment was generated using Snippy v4.4.5 [28] and the AE015929 genome as a reference. Then, iqtree v1.6.12 [29], using model finder [30] and ultrafast bootstrap [31], was used to produce a maximum likelihood phylogenetic tree. A phylogenetic tree of the whole genome SNP was constructed and linked to the gene analysis heat map using the R platform [32,33].

### 2.4. Identification of SCCmec, ACME, and Acquired Antimicrobial Resistance Genes

SCC*mec* were determined for each isolate using the SCC*mec*Finder 1.2 online database [34]. The presence of ACME was searched using the Center for Genomic Epidemiology virulence genes database [35,36]. ACME subtypes were identified after alignment to *S. aureus* USA300 strain FPR3757 (GenBank accession number CP000255) and visualized using the Artemis and BLAST online tools [37,38]. In cases where repeat sequences and mobile elements caused contig break during the assembly, ACME elements scattered in multiple contigs were manually reassembled using BLAST and the ISfinder online database [39]. The online CLUSTAL Omega tool was then used to perform multiple sequence alignments to detect variations in sequences of ACME elements [40]. ResFinder tool from the CGE server was used in this study to detect the acquired antimicrobial resistance genes and their specific location on the sequence [41,42]. The Comprehensive Antibiotic Resistance Database (CARD) (https://card.mcmaster.ca/home (accessed on 20 October 2020)) was also used to detect the presence of putative antibiotic resistance genes using the resistance gene identifier (RGI) tool [43]. 

### 2.5. Antibiotic and Copper Susceptibility

The susceptibility of *S. epidermidis* isolates to copper sulfate (CuSO4) (Sigma-Aldrich, Dorset, UK) was determined by disc diffusion testing. Several colonies from overnight TSA cultures were collected with a sterile loop and resuspended into Mueller Hinton broth (Oxoid, UK) to OD_600_ = 0.5. A Mueller Hinton agar (MHA) plate was inoculated with the culture using a sterile swab in a rotating device to obtain uniform growth. Sterile filter disks cut from filter paper (Whatman, Sigma-Aldrich, St Louis, MO, USA) were impregnated with 20 µL of 1M CuSO_4_ and allowed to dry for 15 min. Filter discs were then firmly applied to the surface of the MHA plate and incubated overnight at 37 °C. Copper susceptibility was determined using the epidemiological cutoff values (ECOFF) [44]. For antibiotic susceptibility, antibiotic discs (BioMérieux and Liofilchem, Nurtingen Germany) for this study were placed on the inoculated MHA plates using sterile forceps. Within 15 min, the plates were incubated at 37 °C for 18–24 h. The antibiotics were selected according to CLSI standard as follows: penicillin G (P 10 mg), amoxycillin/clavulanic acid (AMC 20 mg), oxacillin (OX 1 mg), erythromycin (E 10 mg), cefoxitin (FOX 30 mg), ciprofloxacin (CIP 5 mg), gentamicin (CN 10 mg), clindamycin (DA 2 mg), rifampicin (RD 5 mg), chloramphenicol (C 30 mg), tigecycline (TGC 30 mg). *Staphylococcus aureus* ATCC 25923 was used as a control. For vancomycin and teicoplanin, minimum inhibitory concentrations (MIC) were determined by broth microdilution test, using the Clinical and Laboratory Standard Institute (CLSI) standard [45].

## 3. Results

### 3.1. Comparative Phylogenetic Tree Analysis

**Figure 1:**
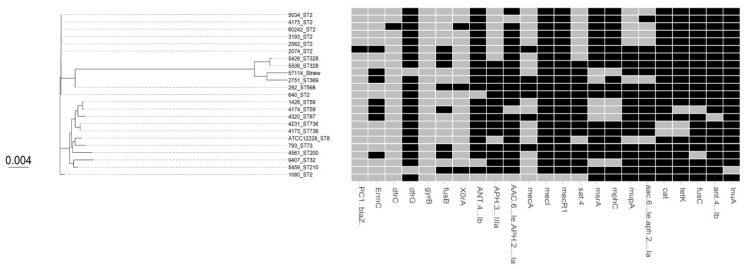
Maximum likelihood phylogenetic tree constructed using core genome alignment from the 24 *S. epidermidis* clinical isolates. The phylogenetic tree is annotated with the isolate’s sequence type ST. *S. epidermidis* ATCC 12228 as a reference strain (GenBank accession number: AE015929).

Multilocus sequence typing (MLST) from whole genome data showed that 33.3% (*n* = 8) of our *S. epidermidis* isolates belong to ST-2 clustered in one branch of the tree (Table 1, Figure 1). ST-2 is the most predominant MDR sequence type worldwide. The remaining MLST were miscellaneous with random distribution throughout the tree (of which two were ST-59, two were ST-328, and two were ST-736) (Figure 1). Three isolates belong to a novel ST, of which two appear to be single locus variants. Out of the 24 *S. epidermidis* strains, 92% were methicillin-resistant *S. epidermidis* (MRSE) harboring the *mecA* gene. Most (81%) of the MRSE isolates carry mainly a type IV SCC*mec* (Table 1). The remaining two MSSE isolates lack *mecA* gene. With the exception of one isolate (2074), all isolates carry PC1 (*blaZ*) gene encoding penicillin resistance. COMER-like element in *S. epidermidis* seems to be larger than COMER in *S. aureus* with additional type I restriction modification system and arsenic resistance (*ars*) operon and exhibited a highly conserved structure in all isolates [3]. However, WGS showed that our *S. epidermidis* lack this composite COMER-like element downstream of SCC*mec*-IV, unlike the COMER element in USA300, with the exception of two isolates (5506 and 292) with *merA* only and one isolate (4526) with *merB* only (Figure 1).

### 3.2. SCCmec and ACME Types 

Types of SCC*mec* are shown in Table 1. The content of *mec* gene complexes and multiple *ccr* gene complexes resulted sometimes to contradicting predictions of the SCCmec type in most isolates. All our isolates harbor the arginine catabolic mobile element (ACME), including both MRSE and MSSE strains. As previously described, ACME types are based on their composition of either one or more of the *opp3*, *kdp*, and *arc* operons as shown in Table 1 [8,10,45,46,47]. In our collection, the vast majority of *S. epidermidis* isolates (67%) belong to ACME type I with ST-2 (*n* = 8)**,** ST-328 (*n* = 2), ST-59 (*n* = 1), ST-new (*n* = 2), ST-73 (*n* = 1), ST-200 (*n* = 1), and ST-598 (*n* = 1). ACME type-II was seen in three isolates (ST-369, ST-8, and ST-59), and ACME type-III in one isolate (ST-new). ACME type-V was seen in five isolates (ST-736 (*n* = 2), ST-87 (*n* = 1), ST-32 (*n* = 1), and ST-210 (*n* = 1)). Unlike COMER-like elements, ACME elements are highly variable in composition, as these are flanked by variable internal direct repeats allowing rearrangement of genes in modules [10]. Upon assembly of ACME in our isolates using the published DRs (A, B, and C) [8,48,49], four different composite islands were constructed (Figure S1, Supplementary Materials) with variable sizes. In addition, eight isolates carrying the ACME-I were located downstream of the SCC*mec*-IV, with *cop* and *ars* genes sandwiched in between and flanked by DR_A and B, respectively, at the 5′ end in *orfX.*

It was observed that partial deletion of SCC*mec* type IV occurred in 2 out of 24 isolates, 1426 and 9407, in which class B *mec* gene complex and composite *ccrA4* were deleted. Deletion of ACME (type IIa) in 4174 (ST-59) was also observed. These rearrangements indicate the high genomic plasticity of *S. epidermidis* particularly in the SCC*mec* and ACME region compared to other regions. This observation has also been noted in in vitro studies where ACME have highly variable structures with 100 times higher excision frequency found for the SCC*mec* elements [3,19].

To examine the relation of our collection of *S. epidermidis* from Oman and the other published *S. epidermidis* strains, the core genome phylogenetic tree was then expanded to include a total of 58 isolates from the Aberdeen, the United Kingdom showed a large clustering of ST-2 lineage [3].

### 3.3. Antimicrobial Susceptibility and Virulence Conferring Genes 

**Figure 2:**
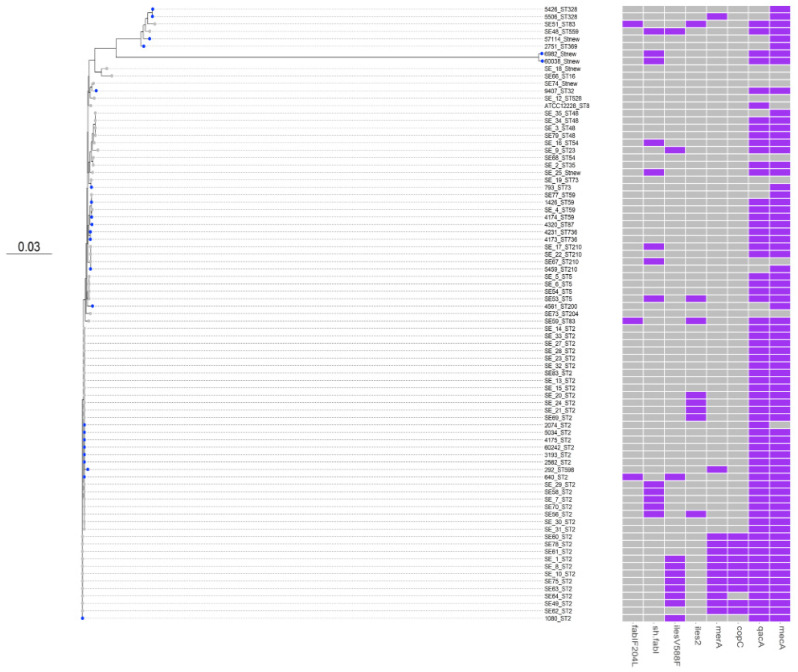
Core genome phylogenetic tree of *S. epidermidis* strains genomes from Aberdeen, UK. Maximum likelihood tree of the 24 isolates from Oman (Table 1) (isolates in marked in blue) and the data set from Aberdeen of 58 isolates (in gray) (NCBI BioProject number: PRJNA574294). As in Figure 1, the tree is annotated with the isolate’s sequence type (ST). Biocide’s tolerance genes (*qacA/B*, *fabI*) and an additional *fabI* allele derived from *Staphylococcus haemolyticus* (*sh-fabI*) are shown. ATCC 12228 is used as the reference strain in this phylogenetic tree (GenBank accession number: AE015929).

Twenty-nine percent of our *S. epidermidis* strains (*n* = 7/24) were *mecA*-negative (Figure 2). In staphylococcal spp., the cefoxitin 30-μg disk was used to predict the *mecA* status, and thus to predict methicillin resistance. Our data were interpreted according to the screening breakpoints published by the CLSI for coagulase-negative staphylococci (CoNS). Inhibition zone diameters for *mecA*-positive isolates were <28 mm for 80% of *S. epidermidis*, indicating methicillin resistance, whereas the remaining 20% *mecA*-positive strains were cefoxitin-sensitive (*n* = 4). Two out of 24 isolates showed oxacillin susceptibility, both of which are *mecA* positive and cefoxitin susceptible. This observation might suggest that *mecA* may be expressed variably in these isolates. Only one strain that was *mecA*-, *mecI*-, and *mecR*-negative showed cefoxitin resistance. Previous studies reported similar findings and suggested that the cefoxitin screening breakpoints for methicillin resistance in CoNS need to be adjusted [50].

In one isolate (2072-ST-2), the *blaZ* gene conferring resistance to penicillin was not detected. However, this isolate lacks the *mecA* gene (coding for altered penicillin-binding protein) and yet showed resistance to penicillin and other beta-lactams. In addition, this isolate is found within a cluster of *blaZ*-positive isolates of the same ST-2, suggesting a possible loss of *bla-Z*. 

Out of 24 isolates, only one strain (60038), which has a mutation in the *rpoB* gene (D471G), showed rifampicin resistance (Table 2 and Supplementary Figure S1). Resistance to ciprofloxacin was found in 14/24 isolates. Mutations in the *gyrA* (S84L) and *parC* (S80Y) gene, which have been described for the USA300 lineage, were present in these resistant isolates. Overall, for the remaining antibiotics, we found a good phenotype-to-genotype correlation for antibiotic susceptibility data. For instance, the presence of macrolide efflux gene (*msrA*) and *emrC* correlates well with the susceptibility phenotype in our isolates as shown in Table 2 and Figure S2 in the Supplementary Materials. 

### 3.4. Biocides Susceptibility

WGS sequencing results enabled the identification of all biocide resistance genes in the 24 *S. epidermidis* isolates (Figure 2). All of our *S. epidermidis* isolates carry a wild type *fabI* gene with no substitutions. The *fabI* gene encodes an enoyl-acyl carrier protein reductase (ENR), which is essential for fatty acid synthesis in bacteria. Isolate (640-ST-2), which carries F204L amino acid substitution resulting in reduced susceptibility to the antiseptic triclosan, is an exception. Moreover, two isolates (60038 and 6982, ST-new) carry an additional copy of *sh*-fabi allele, which is believed to be derived from *Staphylococcus haemolyticus* and results in increased resistance to triclosan [51]. 

Out of 24 isolates, six isolates carry the *mupA* gene coding for mupirocin resistance. Two isolates carry the isoleucyl-tRNA synthetase mutation V588F conferring mupirocin resistance. However, *ileS2* (conferring high level of resistance to mupirocin) was absent in all of our *S. epidermidis* isolates. The carriage of *qacA*, *qacB* and *qacC* genes causing reduced susceptibility to quaternary ammonium compounds was 16.6%, 8.3%, and 12.5%, respectively. 

### 3.5. Copper Susceptibility

**Figure 3:**
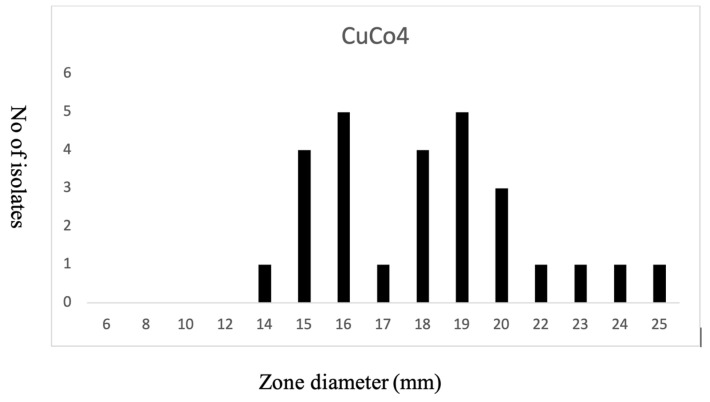
Susceptibility profile of *S. epidermidis* to copper. Disc diffusion inhibition zones were defined for 24 *S. epidermidis* isolates when exposed to 10 µL of 1M stock (1 µg/µL) of copper sulfate (CuSo4).

A phenotypic analysis of copper susceptibility was determined using disc diffusion test on our local *S. epidermidis* isolates (Figure 3). ECOFF was determined based on the normal distribution of the susceptibility, as breakpoints for metal susceptibility is not available [44]. *copR* was present in two strains (793 ST-73 and 5459 ST-210). Phenotypically, these two strains were copper-sensitive. *copAZ* with a second copy was present in all strains except five strains where *copAZ2* was missing. All *S. aureus* species carry a putative copper-sensitive operon repressor (CsoR) -*copA/copZ* operon that is autoregulated by copper-sensitive transcritional repressor (CstR) and appears to encode at least two CsoR-like proteins [52]. Similarly in our collection, all *S. epidermidis* carry copper-resistance-encoding genes, even in the absence of COMER-like elements. This might provide a fitness cost to *S. epidermidis* under high selective pressure environment, as in the hospital settings. 

## 4. Discussion

*S. epidermidis* have successfully emerged from a skin commensal to potentially pathogenic bacteria, owing to its plasticity and increasing capacity of acquiring a reservoir of resistance genes. Research in *S. epidermidis* genomics confirms the high likelihood of spread of the multidrug resistance ST-2 lineage worldwide as well as in our local isolates, where about one third of *S. epidermis* isolates belong to ST-2 [3,4]. The dissemination of MDR ST-2 lineage has been linked to SNPs in the *rpoB* gene, allowing its persistence [4], with possible co-occurrence of the highly stable COMER-like element in *S. epidermidis* clinical isolates [3]. In *S. aureus*, the COMER element enhances its survival in the host by amelioration of the survival capabilities and as a result the infection remains hidden from the immune system [12,13]. However, in our collection, the COMER-like element was not found in any of the isolates, despite its stability in other global lineages. This observation could suggest that the COMER-like element might not commonly occur in our region. All of our *S. epidermidis* isolates carry ACME of various types (I, II, III, and V), which was described initially in oral isolates of *S. epidermidis* and later in bloodstream infections [3,11]. This observation is similar to the epidemiology described in a global ST-2 lineage of Aberdeen, UK [3,53]. Therefore, reduced susceptibility to copper observed in some isolates cannot be contributed by either SCC*mec* alone or ACME or both. In addition, reduced susceptibility to copper was seen in eight isolates belonging to various ST types but predominantly ST-2 (*n* = 4), among others: ST-59 (*n* = 1), ST-328 (*n* = 1), ST-87 (*n* = 1), and ST-736 (*n* = 1). Five out of 8 *S. epidermidis* isolates with reduced copper susceptibility carry ACME-type I. ACME type-II was found in one isolate only. The remaining three belong to ACME type-V (*n* = 2), which is characterized by presence of all three gene clusters: *arc*, which encodes an arginine deaminase pathway; *opp3*, which encodes an oligopeptide permease ABC transporter; and *kdp*, which encodes a potassium ABC transporter [10]. 

ACME elements have been described previously to be more commonly associated with MSSE compared to MRSE [3,46,47,54]. To the contrary, in our collection, 29% of ACME harboring isolates were MSSE and the remaining 71% were MRSE. The relationship between these ACME types and copper susceptibility cannot be inferred at this stage as ST-2 and ACME type I are the predominant genotypes in our limited collection of isolates and a larger sample size will be needed to draw a conclusion. Furthermore, reduced copper susceptibility could well be due to the ubiquity of copper resistance gene clusters in the core chromosome, as it has a fitness advantage to the persistence and evolution of *S. epidermidis* species. Previous studies using infection models have demonstrated that several pathogens have evolved to control excess intracellular copper by either efflux pumps or sequestration as virulence mechanisms [17,18,55,56,57].

In this study, ST-2 *S. epidermidis* clinical isolates showed a lower antibiotics resistance profile compared with the global patterns of some hospital-adapted lineages with a pandrug resistance profile [4]. Apart from the intrinsic resistance to beta-lactams in MRSE, our collection of *S. epidermidis* spares a sensitivity phenotype to several antibiotic groups, including vancomycin, teicoplanin, rifampicin, chloramphenicol, and tigecycline, that have a high level of agreement with their respective genotypes (Figure S2, Supplementary Materials).

In addition to heavy metals, *S. epidermidis* have adapted to biocides commonly used in hospital settings such as quaternary ammonium compounds and triclosan, as well as decolonization antibiotics including mupirocin. Genetic determinants of resistance to mupirocin have been identified (*ileS2*, V588F IleS) and/or triclosan (*sh*-fabI, F204L in *FabI*) in 65% of *qacA S. epidermidis* isolates [53]. *qacA*, *qacB*, and *qacC* encode efflux pumps for a variety of lipophilic cations and are strongly associated with reduced microbial susceptibility to chlorhexidine, which is the most commonly used biocide in the community as well as hospital decontamination and infection control, with applications ranging from mouthwashes to impregnated catheters and skin/mucosal surface decontamination in intensive care units (ICUs). Notably, in a number of cases, mupirocin resistance genes were colocated with *qacA* on variants of known MRSA mobile elements, raising the possibility of horizontal transfer of multidrug resistance genes between *S. epidermidis* and *S. aureus*. However, previous studies have demonstrated the low fitness cost of *ielS*-V588F mutations, as it is consistent and is associated with low burden [58]. However, our study clearly showed high carriage of biocide resistance genes among ST-2 isolates along with MDR and metal resistance genes that are carried on MGEs. Our observation goes in line with the increasing concern from genomics and phylogenetic tree analyses of previous studies—that MDR hospital-adapted lineages consistently carry chlorhexidine, mupirocin, and triclosan-resistance-conferring genes, which is concerning and necessitates further action [59].

In conclusion, in this work, we identify a common prevalence of highly variable ACME elements in different hospital STs of *S. epidermidis* in Oman and high carriage of biocide resistance conferring genes, thus strongly suggesting the hypothesis that ACME types evolved from closely related STs.

## Figures and Tables

**Table 1:** *Staphylococcus epidermidis* isolates.

Isolate	Date	MLST	SCC*mec* Type	ACME Operons & (Type)
5034	November 2018	2	IV (2B)	*arc* & *opp3* (I)
4175	November 2018	2	IV (2B)	*arc* & *opp3* (I)
60242	August 2018	2	IV (2B)	*arc* & *opp3* (I)
3193	August 2018	2	IV (2B)	*arc* & *opp3* (I)
2562	August 2018	2	IV (2B)	*arc* & *opp3* (I)
2074	September 2018	2	-	*arc* & *opp3* (I)
5426	December 2018	328	IV (2B&5)	*arc* & *opp3* (I)
5506	November 2018	328	V (5C2)	*arc* & *opp3* (I)
57114	July 2018	new	Iva (2B)	*arc* & *opp3* (I)
2751	October 2018	369	IVa (2B)	*arc* (II)
292	January 2019	598	IV (2B&5)	*arc* & *opp3* (I)
640	October 2018	2	-	*arc* & *opp3* (I)
1426	October 2018	59	-	*arc* & *opp3* (I)
4174	November 2018	59	Iva (2B)	*arc* (II)
4320	November 2018	87		*kdp, arc* & *opp3* (V)
4231	November 2018	736	Iva (2B)	*kdp, arc* & *opp3* (V)
4173	October 2018	736	Iva (2B)	*kdp, arc* & *opp3* (V)
793	December 2018	73	-	*arc* & *opp3* (I)
4561	November 2018	200	-	*arc* & *opp3* (I)
9407	September 2018	32	-	*kdp, arc* & *opp3* (V)
5459	January 2019	210	V (5C2)	*kdp, arc* & *opp3* (V)
1080	August 2018	2	II (2A)	*arc* & *opp3* (I)
60038	August 2018	new	Iva (2B)	*opp3* (III)
6982	November 2018	new	-	*arc* & *opp3* (I)

**Table 2:** Minimum inhibitory concentrations (MIC) of *S. epidermidis* isolates were determined using the disc diffusion method. The number of isolates that are susceptible, intermediate, or resistant are shown with the percentages in brackets. Broth microdilution methods were used for vancomycin and teicoplanin MICs. Interpretive categories and zone diameter breakpoints were determined according to CLSI.

Antibiotic	No of Isolates (%)		
	Susceptible	Intermediate	Resistant
Penicillin G (P)	0 (0%)	-	24 (100%)
Amoxycillin/clavulanic acid (AMC)	14(58%)	-	10 (42%)
Oxacillin (OX)	2(8%)	-	22 (92%)
Erythromycin (E)	2 (8%)	0 (0%)	22 (92%)
Cefoxitin (FOX)	4 (16%)	-	20 (83%)
Ciprofloxacin (CIP)	9 (38%)	1 (4%)	14 (58%)
Gentamicin (CN)	13 (54%)	1 (4%)	10 (42%)
Clindamycin (DA)	13 (54%)	0 (0%)	11 (46%)
Rifampicin (RD)	23 (96%)	0 (0%)	1 (4%)
Chloramphenicol (C)	22 (92%)	0 (0%)	2 (8%)
Tigecycline (TGC)	24 (100%)	-	0 (0%)
* Vancomycin (VA, MIC)	24 (100%)	-	0 (0%)
** Teicoplanin (TEC MIC)	24 (100%)	-	0 (0%)

* For vancomycin, DD does not differentiate between susceptible, intermediate, or resistant CONs all give similar zone of inhibition. ** Teicoplanin DD interpretive criteria were not re-evaluated concurrent with revaluation of vancomycin DD interpretive criteria. Therefore, the ability of these teicoplanin interpretive criteria to differentiate teicoplanin-intermediate and teicoplanin-susceptible strains is not known.

## Data Availability

Whole genome sequences as draft contigs are available upon request from the corresponding author. The WGS data will be uploaded in GenBank after finishing the project.

## Supplementary Materials

The following are available online at https://www.mdpi.com/article/10.3390/microorganisms9091824/s1, Figure S1: Schematic representation of ACME-I in *S. epidermidis.*, Figure S2: Maximum likelihood phylogenetic tree constructed from WGS data and heat map for the distribution of antibiotic resistance genes of 24 *S. epidermidis* isolates. Table S1: WGS data of *S. epidermidis*: coverage, trimmed reads, taxonomic distribution, and assemblies. Table S2: Antibiotics, copper, and biocide genotype and phenotype results.
