# Supplementary material for: Whole Genome Sequencing of Methicillin-Resistant Staphylococcus epidermidis Clinical Isolates Reveals Variable Composite SCCmec ACME among Different STs in a Tertiary Care Hospital in Oman
Source: Microorganisms. 2021 Aug 27;9(9):1824. doi: 10.3390/microorganisms9091824 (PMC8466944; doi:10.3390/microorganisms9091824)
Supplement: Supplementary file 1 [file microorganisms-09-01824-s001.zip › Supplementary files_Figure S1.pdf]

Supplementary file1

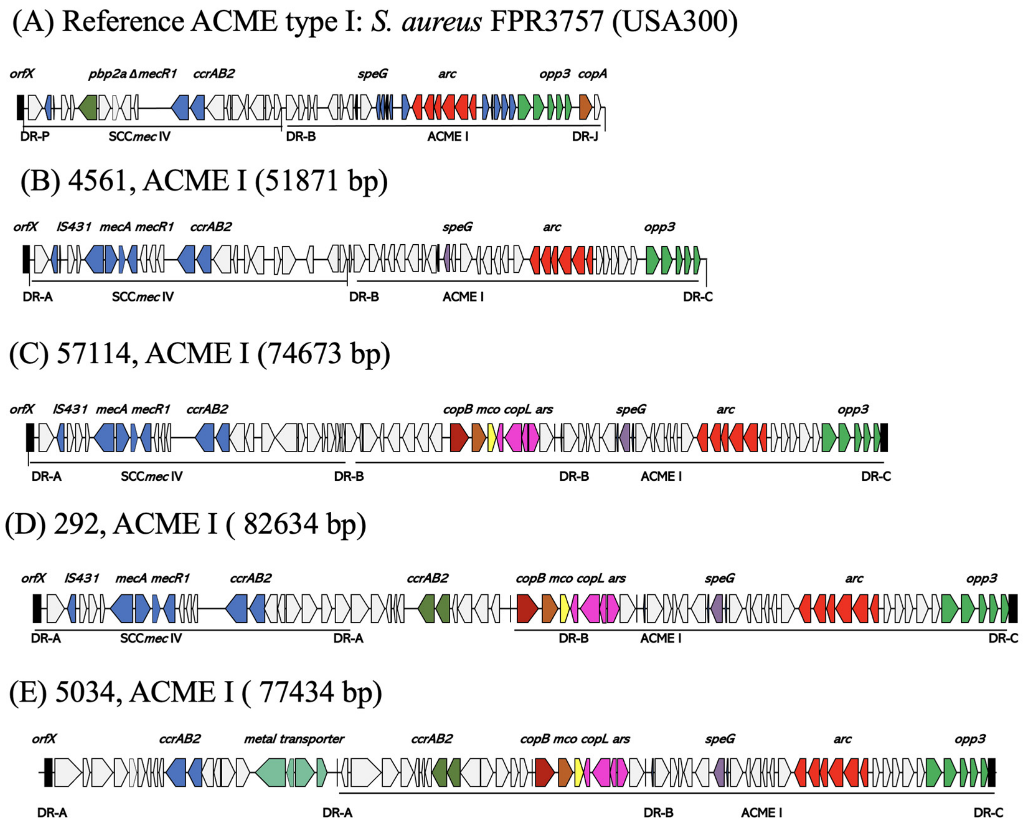

Figure S1. Schematic representation of ACME-I in *S. epidermidis*. comparative maps of ACME-I composite island in 4 ST-200, ST-new, ST-598 and ST-2 *S. epidermidis* isolates. The size of each ACME composite island is indicated after the strain names. Four distinct ACME-I composite island were defined according to the additional modules in these elements (B-E). Each gene or group of genes of interest is shaded in color; *arc* (red) and *opp3* operon (light green), *speG* (light purple), *copB* (Maroon), *mco* (orange), *copL* (ornage), *ars* operon (pink), *mecA*, *mecR1*, *IS431*, *IS1272*, and *ccrAB2* (dark blue). The direct repeat (DR) sequences DRA, DR-B, DR-C are indicated.
