# Supplementary material for: Whole Genome Sequencing of Methicillin-Resistant Staphylococcus epidermidis Clinical Isolates Reveals Variable Composite SCCmec ACME among Different STs in a Tertiary Care Hospital in Oman
Source: Microorganisms. 2021 Aug 27;9(9):1824. doi: 10.3390/microorganisms9091824 (PMC8466944; doi:10.3390/microorganisms9091824)
Supplement: Supplementary file 1 [file microorganisms-09-01824-s001.zip › supplementary file_Figure S2.pdf]

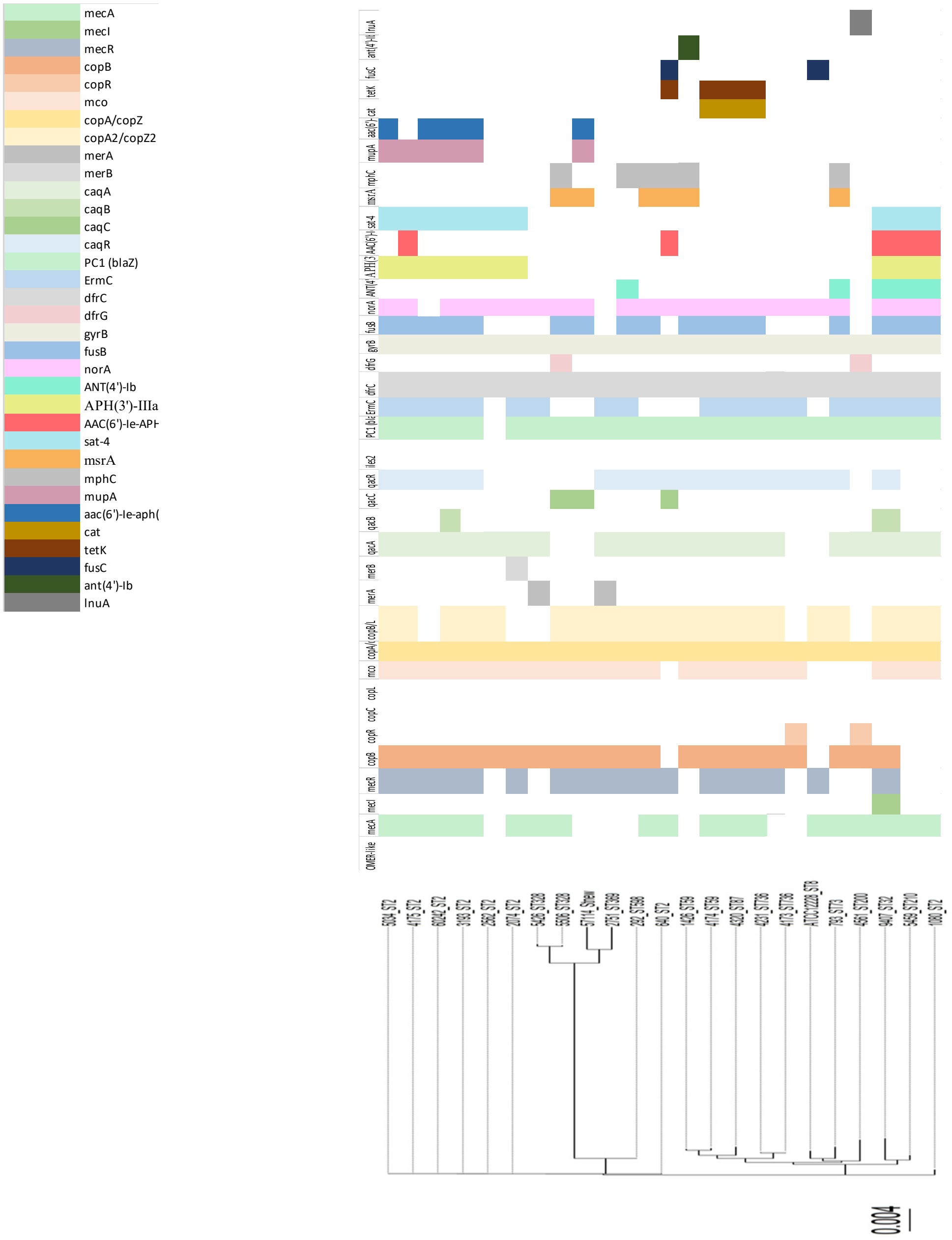

Figure S2. Maximum likelihood phylogenetic tree constructed from WGS data and heat map for the distribution of antibiotic resistance genes of 24 *S. epidermidis* isolates. The phylogenetic tree is annotated with the isolate's number and sequence type (ST). Colored boxes illustrates the distribution of antibiotic resistance genes as shown in the image key on the left hand side. Absent genes are shown in white boxes. ATCC 12228 is used as reference strain in this phylogenetic tree (GenBank accession number: AE015929).
